# Atypical Presentation of Granulomatosis with Polyangiitis

**DOI:** 10.5334/jbsr.1623

**Published:** 2018-11-01

**Authors:** Davide Giordano, Lucia Dardani, Angelo Ghidini

**Affiliations:** 1 Azienda USL-IRCCS di Reggio Emilia, Istituto di Ricovero e Cura a Carattere Scientifico, IT

**Keywords:** granulomatosis, vasculitis, Wegener, polyangiitis, c-ANCA, malignant lesion, mastitis, crusting rhinitis

A 45-year-old female came to our attention with a six-month history of ulcerative mass in her right breast. Definitive pathology on breast lesion (Figure 1) documented the presence of a granulomatous mastitis with necrotic foci (asterisk), giant multinucleated cells (circles), and normal breast ducts in transversal (arrow) and longitudinal section (arrowheads) trapped by neutrophilic-eosinophilic infiltrate. Pathologist found no signs of malignant lesions. During the following months, the patient developed worsening cough, shortness of breath, nasal obstruction with hematic discharge, and hearing loss. Chest computed tomography (Figure 2) showed marked wall thickening of the right main bronchus (asterisk), stenosis of the left main bronchus (black arrowheads), and segmental atelectasis in the upper lobe of the left lung (white arrowheads). Paranasal sinuses computed tomography (Figure 3) showed a perforation of the nasal septum (asterisks), absence of the anterior half of the left inferior turbinate (white arrows), and atrophy of the upper lateral cartilages (white arrowheads). Fiberoptic rhinopharyngoscopyscopy documented the presence of extensive crusting rhinitis. Blood tests revealed high levels of antineutrophilic cytoplasmic antibodies with cytoplasmic pattern (c-ANCA). These findings were consistent with diagnosis of granulomatosis with polyangiitis. The patient was put under oral prednisone 1 mg/kg daily and cyclophosphamide monthly pulse therapy, with progressive improvement of breast and nasal lesions. She has currently reached a clinically stable NYHA Class II and is treated with prednisone 15 mg daily, methotrexate 15 mg weekly, and n-acetilcysteine 600 mg daily. She needs periodic endoscopic dilatations of bronchial stenosis.

**Figure 1:**
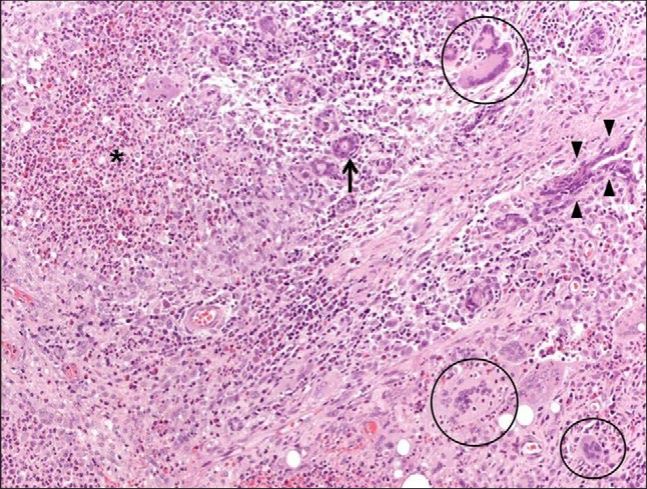
Microphotograph (magnification: 40X) showing a granulomatous mastitis. Mammary parenchyma is characterized by the presence of dense necrotic foci (asterisk), giant multinucleated cells (circles), and normal breast ducts in transversal (arrow) and longitudinal section (arrowheads), trapped by neutrophilic-eosinophilic infiltrate.

**Figure 2:**
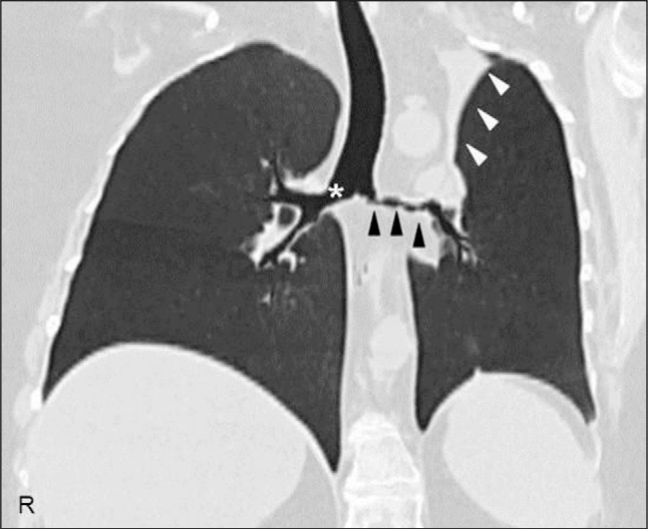
High-resolution computed tomography of the chest, coronal reconstruction, showing a marked wall thickening of the right main bronchus (asterisk), stenosis of the left main bronchus (black arrowheads), and segmental atelectasis in the upper lobe of the left lung (white arrowheads).

**Figure 3:**
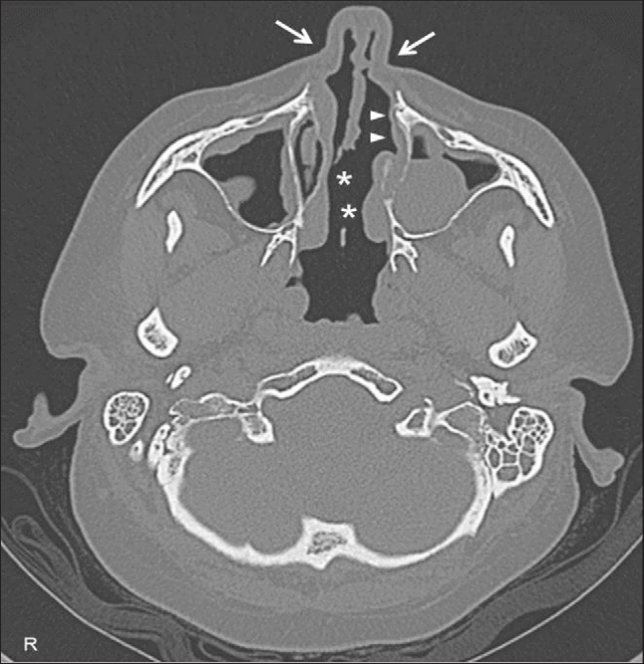
High-resolution computed tomography of paranasal sinuses, axial projection, showing a perforation of the nasal septum (asterisks), the absence of the anterior half of the left inferior turbinate (white arrows), and the atrophy of the upper lateral cartilages (white arrowheads).

## Comment

Granulomatosis with poliangiitis (GPA) is an autoimmune systemic vasculitis of the small vessels. Infectious, environmental, chemical, and toxic triggers have been advocated as etiologic triggers in predisposed people. It is usually associated with high levels of circulating antineutrophilic cytoplasmic antibodies (ANCA), even if positive ANCA serology is not pathognomonic of GPA, especially in the absence of clinical and histological findings of systemic vasculitis. Clinical manifestations of GPA include constitutional symptoms, mucocutaneous manifestations of upper airways, typically given by oral ulcers and crusting rhinitis, and subglottis laryngeal stenosis, lower respiratory tract manifestations, such as pulmonary nodules and infiltrates, bronchial wall thickening, and pleural effusion. Some patients may develop cardiovascular, renal, or central nervous system involvement. To the best of our knowledge, inflammatory mastitis as a presenting symptom of GPA has never be reported, even if cutaneous ulcers, digital infarcts, purpura, although not pathognomonic, are not infrequent complaints of GPA. The histological feature of GPA is characterized by necrosis, focal vasculitis of small veins, arteries, and capillaries, and inflammatory infiltrate composed by neutrophils, lymphocytes, plasma cells, macrophages, eosinophils, and giant multinucleated cells. High resolution computed tomography helps in achieving diagnosis, as it shows typical GPA-related pulmonary findings such as nodules, cavitary lesions, tracheobronchial stenosis, and interstitial disease. Prompt diagnosis plays a capital role because a proper treatment may induce clinical remission, ameliorate the morbidity, and greatly improve the survival. Currently, management of GPA is based on immunosuppressant drugs, such as cyclophosphamide, azathioprine, and methotrexate, monoclonal antibodies such as rituximab. Plasma exchange has been proposed in conjunction with cyclophosphamide for patients with aggressive vasculitic involvement of the kidney [1].

## References

[1] Lutalo, PM and D’Cruz, DP. Diagnosis and classification of granulomatosis with polyangiitis (a.k.a. Wegener’s granulomatosis). J Autoimmun. 2014 Feb-Mar; 48–49: 94–8. DOI: 10.1016/j.jaut.2014.01.028 24485158

